# Job satisfaction and animal welfare at slaughter: A survey of Official Veterinarians in the United Kingdom and Republic of Ireland

**DOI:** 10.1017/awf.2024.43

**Published:** 2025-01-20

**Authors:** Imogen O’Connor, Kelly Gouveia

**Affiliations:** University of Chester, School of Natural Sciences, Faculty of Science and Engineering, Exton Park Campus, Parkgate Rd, Chester CH1 4BJ, UK

**Keywords:** animal welfare, job satisfaction, Official Veterinarian, religious slaughter, slaughter, UK and ROI

## Abstract

In the UK and the Republic of Ireland, Official Veterinarians (OVs) are employed by the Food Standards Agency and the Food Safety Authority, respectively, as legal authorities for both animal welfare and food safety. However, little is known about job satisfaction in this profession which has the potential to impact professionals’ well-being and performance. Moreover, despite animal welfare issues being a reality that OVs witness, we do not yet understand how OVs perceive these issues at slaughter or whether this impacts job satisfaction. We assessed OVs’ perceptions on job satisfaction and views on welfare at slaughter across the UK and ROI, through an online questionnaire with 113 participants, which included socio-demographic information of participants and questions or statements about different aspects of job satisfaction and animal welfare issues at slaughter. While most OVs committed to their work they reported issues that may compromise job satisfaction, such as often experiencing loneliness at work, threatening situations and sleep disorders. Moreover, job satisfaction was often impacted by animal welfare incidents, and conflicts with food business operators were considered one of the greatest barriers to improving welfare at slaughter. There is also the likelihood of professionals’ individual ethical values being challenged since OVs are virtually certain to witness religious slaughter yet disagree with this practice to the extent that they consider it should be banned. We reveal significant challenges associated with the role of OV that justify creation of a support network to assist and safeguard this profession, as well as animal welfare at slaughter.

## Introduction

In accordance with European Union (EU) legislation, Official Veterinarians (OVs) are employed in slaughter plants as a legal authority to provide certification that meat to be included in the human food chain derives from healthy animals, and that unnecessary suffering has not occurred during the slaughter process (Regulation 2017/625 2017). In the UK, OVs work for the competent authority on behalf of the Food Standards Agency (FSA) and for the Food Safety Authority in the Republic of Ireland (ROI) to ensure that slaughterhouses are compliant with all legal requirements detailed in national and international legislation on food safety and animal welfare (Food Safety Authority Ireland 2021-2025; Food Standards Agency 2020a). For this, a zero tolerance approach is required to be adopted as regards breaches of legislation and all instances of non-compliance must be reported to the respective legal authorities (Food Standards Agency 2020b). Despite this, very little is known about how non-compliances affect OVs’ job satisfaction and personal well-being, particularly when animal welfare incidents occur. This is critical within the meat industry, particularly in the UK, given the general expectation of high standards of welfare and ethical treatment of animals (Royal Society for the Prevention of Cruelty to Animals [RSPCA] 2018). Moreover, since Brexit, the UK has been very vocal in its commitment to implementing higher regulatory standards than the baseline set by the European Union (RSPCA 2018). Therefore, understanding how animal welfare is perceived through the ‘lens’ of an OV is also likely to provide a more accurate insight into the reality of animal welfare at slaughter since OVs, being the legal authority for animal welfare at slaughter, are best placed to pass judgement on the potential welfare challenges that occur at slaughter.

There is a possibility, however, that an OV’s role may not appeal to all professionals since it includes a vast amount of solitary work with unsociable hours with many slaughter plants operating overnight or during the early hours of the morning. OVs must also meet their contractual obligations; their signature must be present on all relevant documents certifying that the meat produced for the human food chain meets national and international requirements for food hygiene and animal welfare (Regulation 854/2004 2004). This can lead to frequent overtime obligations in order to ensure OVs fulfil the role’s administrative requirements (Väärikkälä *et al.* 2020). Also, it is a role that entails observing the killing of animals by both stunning and ritual slaughter, and the possibility of witnessing animal welfare insults during the slaughter process (Humane Slaughter Association 2006). It is likely to contrast with most veterinarians’ expectations since individuals often enter this profession with the intention of using their clinical skills to promote animal health and welfare (Wensley *et al.* 2020). Therefore, the slaughterhouse environment can be challenging for professionals to overcome due to this discrepancy between professionals’ expectations and the reality of life as an OV (Wojtacka *et al.* 2020). Indeed, moral dilemmas are known to arise in this profession as a result of this mismatch. An example of this would include attitude towards emergency slaughter (Magalhães-Sant’Ana *et al.* 2016) whereby the veterinarian faces the scenario of an animal having been transported to slaughter despite severe lameness or injuries affecting mobility being clearly evident. Another such example causing moral stress is the reality of often having to work alone in this profession and make difficult decisions without appropriate support (Väärikkälä *et al.* 2020). Moreover, low levels of satisfaction and work-related stress in the veterinary profession have often been linked to withdrawal behaviours, such as absenteeism, burn-out, and mental illness (Kersebohm *et al.* 2017; Moir & Van Den Brink 2020), all of which inevitably impact upon job performance. Therefore, it is essential to determine whether this is the case in the OV profession.

There are many elements to the slaughter process that OVs have a legal responsibility to uphold. This includes reporting areas they may consider poses a risk to pest infestation, carcase contamination, or even structural and procedural issues that put animals at risk of injury or unnecessary suffering prior to slaughter (Food Standards Agency 2020b). Non-compliances need to be communicated to the slaughterhouse’s food business operator (FBO) for them to respond accordingly and report the issue to the legal authorities. However, in certain circumstances, this may lead to FBO-OV conflicts, particularly when correction of non-compliances requires the implementation of new infrastructures likely to come at a price that may lie beyond the range the FBO would be willing to invest (Akporhuarho & Achoja 2017). This could be particularly pertinent in cases of smaller businesses that require new infrastructures (e.g. improved lairage design or stunning boxes) and could certainly lead to work-based conflicts and tensions between OVs and the FBO (Mari *et.al* 2013; Barter 2014). Moreover, it is possible that specific OV demographics could impact their perceptions as regards job satisfaction and animal welfare at slaughter, even contributing to OV-FBO conflicts. Clinical experience and/or age are of particular significance since young and less-experienced veterinarians could feel intimidated as FBOs generally tend to be experienced in the meat industry and potentially less inclined to acknowledge non-compliances from recent OV graduates, resulting in work conflicts (Kaskela *et.al* 2019; Wojtaka *et.al* 2020). It is important, therefore, to investigate whether age or professional experience could act as potential risk factors for job satisfaction or personal well-being in the profession.

A recent study by Gomez-Neves *et al.* (2023) across European countries found training to be one component of job satisfaction that was consistently deemed deficient by OVs, in particular concerning matters of food hygiene and animal welfare requirements, leading to them subsequently feeling unprepared to fulfil their duties. This finding is especially concerning given OVs’ legal responsibility as regards public health and animal welfare and warrants further research to determine where and how training can meet the professional needs of OVs. This is also likely to cause work-related stress (Väärikkälä *et al.* 2020) potentially impacting upon professionals’ job satisfaction. Moreover, it is unknown whether implementing refinements to animal welfare in the meat industry leads to compliance in official inspections. Of particular interest is the success of closed circuit television (CCTV) in terms of improving animal welfare at slaughter – currently a legal requirement for UK slaughterhouses. This is important to ascertain as it has obvious implications for animal welfare and could impact on OV job satisfaction. OVs have the potential to provide valuable insight into this given that they are authority responsible for taking legal action and reporting any non-compliance to the relevant authorities.

Furthermore, to the authors’ knowledge, there have been no studies offering an assessment of OVs’ perceptions regarding animal welfare at slaughter, not to mention overall job satisfaction in the UK or ROI. To assess this, we designed a questionnaire that investigated several aspects of job satisfaction, including job commitment, relationship with the FBO, and the work/life balance. Secondly, the questionnaire focused on assessing OVs’ perception of animal welfare at slaughter via an examination of their views concerning various aspects of animal welfare, including, opinions on ritual slaughter, the effectiveness of recent legislation requiring CCTV, and their level of satisfaction with the FBO on compliance with welfare matters. Finally, we sought to explore the effect of demographics, such as age, professional experience, sex and species of animal worked with on respondents’ opinions on job satisfaction and animal welfare at slaughter.

## Materials and methods

### Ethical approval

This study gained approval from the research ethics committee of the Department of Biological Sciences at the University of Chester, UK.

### Questionnaire survey

An electronic survey was created to assess job satisfaction and well-being of OVs working in the UK and the ROI and their perceptions on animal welfare at slaughter. The survey has been reported according to CROSS (Checklist for Reporting of Survey Studies; Sharma *et al.* 2021). Methods were adapted from Väärikkälä *et al.* (2020) to acquire information on the OVs’ level of job satisfaction. The questionnaire included a participant information sheet where the purpose of the study, voluntary participation, and confidentiality were outlined. Additionally, it was also established that by completing the questionnaire the respondent consented to participating in the study. Only current professionals employed as OVs were recruited via voluntary participation, the aim being to capture the current reality of life as an OV. In total, 129 surveys were emailed to OVs working in slaughterhouses for a private company in the UK (Eville and Jones, Ltd) and the Department of Agriculture Food and Marines in the ROI. The survey remained opened for six weeks between November and December 2020, and reminder emails sent weekly to participants to maximise uptake. No follow-up study was carried out to determine reasons for a lack of response to the survey. A response rate of 87.6% (n = 113) was achieved which was largely representative of our original population sample.

The questionnaire comprised of 19 close-ended, multiple-choice questions covering the following topics: (1) demographic information; (2) challenges in the work environment that may impact job satisfaction; and (3) perception of the systems in place to safeguard animal welfare at slaughter. The survey utilised a Likert scale approach with multiple categories from which respondents could choose to indicate the extent of their opinions, attitudes, or feelings about a particular issue (Nemoto & Beglar 2014). This included assessing the participant’s level of agreement with the statements provided within the questionnaire or level of satisfaction with a particular aspect of their job role.

### Statistical analysis

All statistical analyses were conducted using SPSS® statistical software (IBM SPSS® Statistics v26.0). Initially, a descriptive exploratory analysis was carried out to examine general trends in test responses across respondents for each question. For dependent variables that were ordinal, Kruskal-Wallis or Mann-Whitney tests were used to examine associations between the independent variable (species, sex, age, professional experience) and the dependent variable, i.e. the participants’ responses (du Prel *et al.* 2010). Non-parametric statistical analysis was carried out as the Kolmogorov-Smirnov test of normality revealed that the data were not normally distributed (*P* < 0.05 for all). Where the dependent variable outcome was categorical, non-parametric Pearson’s Chi-squared statistical tests for association were applied to examine this association (Balakrishnan *et.al* 2013). Associations between location of the slaughterhouse and participant responses were not assessed in this study due to the very small sample size of participants working outside of UK. A Spearman’s rank test was carried out to assess correlations between sleeping disorders and loneliness at work.

## Results

### Demographic data

The majority of our respondents worked in UK (n = 76; 67.3%), with fewer in the ROI (n = 31; 27.4%), and some working in both countries (n = 6; 5.3%). There was a relatively even split between the sexes in this study, with 58% (n = 66) male and 42% (n = 47) female participants. The majority of participants were aged between 31–40 (n = 36; 31.9%) and 41–50 years (n = 14; 35.4%) of age. Fewer participants were aged up to 30 years (n = 13; 11.5%), 51–60 years (n = 14; 12.4%), and 60+ years (n = 10; 8.8%). More than half of the respondents consisted of veterinarians working with bovines, and in small ruminant slaughterhouses (n = 70; 62%) with a smaller contingent working in swine (n = 22; 19.4%) and poultry (n = 21; 18.6%) slaughterhouses. Most respondents had up to five years of professional experience (n = 61; 58%) with less having between 6–9 years’ experience (n = 15; 13.3%) or 10+ years (n = 37, 32.7%).

### Official Veterinarians’ responses to job satisfaction

The majority of OVs reported that they were very committed to their work (n = 91; 81.3%; Table S1[a]; Supplementary material). Work-life balance was described as reasonable by more than half (n = 70; 61.9%) of the participants, with a minority reporting either unsatisfactory (n = 16; 14.2%) or optimal work-life balance (n = 27; 23.9%). Loneliness at work was reported by 66.4% (n = 68) respondents, with only a third (n = 38; 33.6%) reporting never having experienced loneliness. Most (n = 79; 69.9%) reported at least sometimes suffering from sleeping disorders due to work-related factors. Closer analysis revealed a positive correlation between experiencing loneliness in the work environment and sleeping disorders (Spearman’s correlation = 0.4; *P* < 0.001). Threatening situations in the workplace in the previous 12 months, including incidents in which the individual’s physical health or life had been endangered while fulfilling professional duties or through intimidation in the workplace were reported by approximately a third (n = 36; 31.9%) of professionals. The majority of veterinarians were at least somewhat satisfied (n = 92; 81.5%) that sufficient training in animal welfare had been provided in regards to their role as an OV.

### Official Veterinarians’ responses to perceptions on animal welfare at slaughter

The majority of respondents (n = 88; 77.9%; Table 1[b]; Supplementary material) reported that their job satisfaction was most commonly impacted by animal welfare incidents at least sometimes. Approximately half (n = 56; 49.5%) reported administrative processes to be time-consuming and that they interfered with the ability to conduct thorough animal welfare inspection, while only a minority disagreed with this statement (n = 20; 17.7%). The majority were at least ‘somewhat satisfied’ (n = 92; 81.4%) that a suitable system was in place for animal welfare at their establishment. Variable responses arose as regards the specific locations where the most prominent welfare issues occurred in the slaughterhouse. Unloading accounted for approximately a third (n = 33; 29.2%) followed by more than one of the listed options receiving approximately a quarter (n = 24; 21.2%) of total responses. Just under a quarter of the participants reported issues in the lairage (n = 19; 16.8%), during movement and restraint (n = 19; 16.8%) and during stunning and bleeding procedures (n = 18; 15.9%). Most respondents (n = 99; 87.6%) agreed that they were able to communicate their welfare concerns to the FBO and receive effective compliance. The majority of OVs agreed (n = 95; 84.1%) that ritual slaughter without stunning should be banned in the UK and ROI. Most (n = 79; 70%) were in agreement that CCTV had reduced the frequency of animal welfare non-compliances. The attitudes and willingness of the FBO was perceived as the greatest barrier for improving animal welfare standards at slaughter (n = 44; 38.9%), in conjunction with all of the above options, which included financial constraints, attitudes of FBO, and lack of appropriate legislation (n = 34; 30.1%).

### Associations between Official Veterinarians’ demographic characteristics and their responses

Overall, neither age nor professional experience seemed to impact participants’ responses on job satisfaction, which indicated that OVs’ opinions were consistent across time in the profession (Tables S2, S3; Supplementary material). However, there was a significant association between age of professionals and their perception of their work-life balance (χ^2^ [4] = 18.5; *P* < 0.001). This showed that older participants perceived their work-life balance as being mostly reasonable (31–40 years: n = 24; 66.7% within age group; 41–50 years: n = 26; 65%) or optimal (31–40: n = 7; 19.4%; 41–50 years: n = 10; 25%) in comparison to young professionals up to 30 years of age where, for instance, none reported this as optimal and some even described it as unsatisfactory (n = 5; 38.5% within age group; Table S6; Supplementary material). There was a strong statistical association between age and the degree of loneliness experienced at work (χ^2^ [4] = 14.2; *P* = 0.007). This was because young professionals tended to report feelings of loneliness often or always (up to 30 years: often; n = 5; 38.5%; always; n = 3; 23.1 %) more consistently, whereas older professionals were report this less (31–40 years: often; n = 6; 16.7%; always; n = 1; 2.8%; 41–50 years: often n = 8, 20.0%; always n = 2, 5.0%; 51–60 years: often n = 3; 21.4%; always n = 1; 7.1%; 61+ years: often n = 0; 0%; always; n = 0; 0%; Table S6; Supplementary material). Perceptions on animal welfare at slaughter were not influenced by the age of the respondent (Table S2[b]; Supplementary material), although there was borderline significance seen with area(s) of the slaughterhouse where issues were most encountered (χ^2^ [20] = 31.0; *P* = 0.056). This was because there was a trend towards respondents in the 31–40 years age-group identifying more than one area where the most prominent welfare issues occurred (n = 12; 33.3%), in contrast to other age groups where the most common response was unloading (Table S2[b]; Supplementary material).

Respondents’ sex showed no association the majority of the views on job satisfaction or animal welfare at slaughter (Table S4[a],[b]; Supplementary material). There was, however, a borderline tendency observed for female respondents to report lower levels of satisfaction with training provided by their company (very satisfied: n = 18; 38.3% within sex) compared to males (very satisfied: n = 38; 57.6%; *U* = 1247.5; *P* = 0.054). There was also a significant difference between male and female professionals in admitting that administration processes interfere with animal welfare inspections (*U* = 1,167; *P* = 0.020) with males more willing to strongly agree (n = 14; 21.2%) than females (n = 3; *P* = 6.4%). Similarly, male respondents agreed more strongly with the notion that ritual slaughter should be banned in the UK (n = 38; 57.6%) compared to females (n = 0; 0%; *U* = 1,220.5; *P* = 0.021).

### Official Veterinarians’ responses and their associations with the species slaughtered

Species showed no association with OV responses on job satisfaction or welfare at slaughter (Table S5[a],[b]; Supplementary material).

## Discussion

This study set out to investigate OVs’ perceptions on job satisfaction, as well as assess their views on animal welfare at slaughter in order to provide an insight into the potential issues facing professionals in the UK and ROI. Most professionals revealed themselves to be committed to their jobs, however the work-life balance in particular was found to be less than satisfactory with sleeping disorders and even threatening situations an all-too-common reality. We also found that incidents where animal welfare was compromised had the potential to impact upon OV well-being and job satisfaction, and that responses to these were not influenced by OVs’ demographic factors. Our study emphasises the need to reconsider priorities for OV well-being, as well as targeted strategies to improve retention and satisfaction in this profession.

Here, two-thirds of participating OVs reported loneliness at work, a finding in accordance with previous research on this profession (Väärikkälä *et al.* 2020). Such experiences potentially reflect the nature of the role since the OV is deemed the legal representative for animal welfare and public health in the slaughterhouse and, as such, often works alone and independently whilst occasionally overseeing collaborative tasks with other officials. Interestingly, similar trends have been observed among medical professionals working within the healthcare industry, where loneliness is thought to stem from a mismatch between the employees’ values and the industry requirements, eventually leading to alienation at work and poor job performance and satisfaction (Santas *et al.* 2016). Neither the causes nor the consequences of loneliness were explored in this study, however it is possible that this reflects how loneliness develops in the food safety industry. Further, the greater frequency of loneliness found in the younger demographic group could be further complicated through the lack of established working relationships with colleagues often seen in individuals only recently introduced to their profession (Fritschi *et al.* 2009). Another possibility is that this finding reflects survival bias here (Howe & Robinson 2019), whereby professionals who may have been dissatisfied perhaps quit their job, leaving behind only those professionals that overcame any initial work-based challenges and thus now express greater job satisfaction. This study was also carried out during the COVID pandemic and the social restrictions that were imposed during this period could have further contributed to the sense of social isolation (Lee *et al.* 2020; Wong *et al.* 2020). Implementing further follow-up studies would be useful to confirm whether such findings are consistent in this profession.

Most OVs reported suffering from sleeping disorders which is perhaps unsurprising given the work patterns often associated with work of this type, for example, early morning shifts which are known to disrupt normal circadian rhythms (James *et al.* 2017). Further analyses revealed a strong positive association between loneliness at work and sleeping disorders. A finding that reiterated the notion of loneliness in the work environment leading potentially to job-related stress and sleeping disorders (O’Connor *et al.* 2020; Väärikkälä *et al.* 2020). Further, it is already well known that job-related stress and sleeping disorders can impact mental health, leading to burn-out and various other mental health issues in the veterinary profession (Quedraogo *et al.* 2021; Neil *et al.* 2022). Therefore, this finding is cause for concern, due to potential health consequences. Further research would be valuable, helping clarify the extent to which sleeping disorders affect OVs’ mental health and job performance with implications not only for professionals involved, but also for food safety and animal welfare.

Young professionals most frequently reported work/private life balance as either reasonable or unsatisfactory in this study. Indeed, work conditions in the OV role, particularly long shifts and disrupted sleep patterns have been shown to manifest as lower levels of job commitment, productivity and ultimately job performance (Costa *et al.* 2006). In addition, the likelihood is that younger OVs would tend to receive less financial remuneration in the UK due to often earning a starting salary (Card *et al.* 2012). Collectively, this could potentially leave younger professionals at a disadvantage in terms of job satisfaction. In contrast, older professionals, aged 60 years or older, were more willing to report their work-life balance as optimal. This could be due to professionals at this stage of their career tending to earn closer to the higher end of their pay scale and preferring to work reduced hours (Bell & Rutherford 2013), thereby cultivating a healthier work life balance. It is also possible that this finding could reflect potential survival bias in the profession. Further research is essential to determine how and why working conditions in older demographic populations are optimal to replicate similar outcomes in younger professionals and conserve both satisfaction and retention in this profession. Studies that specifically follow-up individual’s job satisfaction over prolonged periods of time for a more accurate reflection of the reasoning behind the differences found for age in job satisfaction would be highly relevant.

Interestingly, around one-third of OVs taking part in this study reported experiencing threatening situations in the workplace in the last 12 months. This included incidents in which professionals’ felt their physical health or life was endangered whilst carrying out specific duties in the workplace. Indeed, threatening situations may arise from a conflict of interest between the OV and the FBO (or respective representatives or operators), which is likely to occur when the FBO disputes the OV’s decision to take legal action against a non-compliance (Kaskela *et al.* 2019; Wojtacka *et al.* 2020). Incidents where welfare is compromised may also be an important cause for the breakdown of the FBO-OV relationship. A notion supported by the majority of participating veterinarians reporting that the attitudes of the FBO and their willingness or otherwise represented the most significant barrier to improving animal welfare at slaughter. OVs however were mostly satisfied with FBO compliance for animal welfare at slaughter.

No further clarification was sought from OVs concerning the exact root cause of them being threatened or the direct circumstances leading up to the situation they had encountered. This would require further research to clarify where and how breakdown of the OV-FBO relationship occurs and which factors are most likely to lead to this in order to prevent the occurrence of such incidents. OVs’ working relationships may be damaged irreparably by such incidents. Interestingly our study found no link between OVs’ demographic factors and the likelihood of threatening situations arising with FBOs, demonstrating, at least, that age, sex or professional experience are unlikely to account for these incidents.

In this study, job satisfaction was impacted by the compromises to animal welfare that are encountered in the profession. This finding is concerning given the ethical standpoint of veterinarians, particularly as regards their ongoing pledge to ensure animal welfare is prioritised (RCVS 2022). This may create an ethical dilemma, as the veterinarian is acutely aware of their own professional ethical principles, but since welfare incidents may have already occurred, for example those taking place on-farm or during transport, often their only means of intervention is recourse to legal action. Indeed, here, the most commonly reported welfare issues at slaughter were seen during unloading, where incidents such as trauma, disease or dead-on-arrival are encountered during routine inspection procedures. Moreover, welfare incidents encountered at slaughter may result in compassion fatigue, characterised by physical and emotional exhaustion or development of mental health disorders (Newsome *et al.* 2020; Pohl *et al.* 2022). This is widely acknowledged in veterinary clinical practice in response to traumatic events with animals (Newsome *et al.* 2020), and participants’ responses to welfare incidents confirm this could well be the case in the OV profession. Another important consequence of compassion fatigue or secondary traumatic stress is that it can lead to reduced quality of animal care, and in the case of OVs this could mean prevention of future incidents could be compromised if OVs fail to report animal welfare non-compliances. Compassion fatigue is also an important cause of loneliness and feelings of isolation in the workplace (Scotney *et al.* 2015), which is in accordance with most OVs reporting these precise feelings in this profession. Further research is required to determine the mechanisms underpinning development of compassion fatigue in this profession, as well as the relative importance of loneliness and the relationship with the FBO as potential risk factors, to define specific strategies for improving job satisfaction for OVs.

More than half of the OVs that participated agreed administrative tasks interfered with welfare inspections. These tasks are known to take up substantial amounts of time, with OVs often having the greatest proportion of their shift taken up with completing paperwork to ensure compliance with public health safety (Salines *et al.* 2018). This is another finding which is of concern, creating an ethical dilemma due to the expectation that veterinarians should always make the welfare of animals their number one priority. If administrative tasks are becoming increasingly time-consuming, to the point that they are impinging on time needed for welfare checks, support is needed to limit the impact on welfare inspections, whilst continuing to ensure public food safety. Furthermore, most OVs perform animal welfare inspections alone, most likely due to financial constraints (Council of the Association of Government Veterinarians 2019) despite suggestions that it would be of greater benefit for them to operate in pairs. This would not only reduce loneliness and workload but also alleviate the stress of potentially threatening situations as OVs would feel less vulnerable (Väärikkälä *et al.* 2020). Having typical tasks associated with inspection, such as making observations, taking photographs, and writing up notes, performed with a colleague would decrease the surplus of OVs’ administrative work and help ensure that animal welfare inspections were completed more regularly (Väärikkälä *et al.* 2020).

The majority of OVs were in agreement that ritual slaughter without stunning should be banned in the UK and ROI. Yet, currently, Halal slaughter in the UK is mostly (over 80%) carried out with pre-cut stunning, although Shechita slaughter remains an exception since any form of stunning is strictly prohibited by Jewish law (Fuseini *et al.* 2022). Therefore, despite the implementation of this mitigation in religious slaughter, OVs still consider that this practice should be banned. This could be because such mitigations are complex and unfeasible in a number of species meaning OVs are still likely to be exposed to slaughter without stunning on a regular basis (Abdullah *et al.* 2019; Fuseini *et al.* 2019). For example, in cattle, pre-cut stuns are not carried out due to the possibility of the animal dying prior to being bled, an outcome forbidden in Quran law for Halal slaughter (Abdullah *et al.* 2019). However, the species in question was not a factor in OVs’ level of agreement regarding religious slaughter, illustrating that this response was unanimous among most OVs irrespective of animal. Instead, it is possible that OVs might consider religious slaughter to cause unnecessary suffering regardless of any mitigations put in place. Indeed, a study conducted by Fuseini *et al.* (2019) showed that most veterinary students consider slaughter without stunning as painful and likely to cause unnecessary suffering. Moreover, it seems likely that having a veterinary background could dispose officials to consider this practice as inhumane, and our study shows that this is likely to reflect in professionals’ desire for this practice to be abolished. The consequences of this stance give definite cause for concern since it is a reality of the role of OV that religious slaughter will be encountered. This discrepancy between a practice OVs’ consider a breach of welfare yet continue to be exposed to has the potential to cause them a moral dilemma, professional dissatisfaction and, more seriously, have implications for their health, such as burn-out or other mental health conditions.

In this study, FBO attitudes were identified as the most significant barrier to improving animal welfare at slaughter. This finding suggests that the greatest challenges the meat industry faces in implementing animal welfare improvements in slaughterhouses may not only be finance related, as widely acknowledged, but also motivational. Indeed, risk perception is thought to be a crucial factor in influencing food safety behaviour by FBOs (Kaskela *et al.* 2019), so this could well apply to decisions related to improving animal welfare. It is possible that despite welfare training and food businesses having their own animal welfare quality control system in place, through the role of animal welfare officer (WATOK 2015), FBOs may not perceive potential factors for improvements to the welfare in their facilities. Further, while quality control manuals encourage high standards of food hygiene and animal welfare, FBOs are only legally bound to comply with legislative requirements. Accordingly, we found that OVs were mostly satisfied with FBOs achieving compliance with welfare requirements, as this is a legal requirement. However, striving to achieve compliance with current regulations would appear insufficient in ensuring progress as regards sustained improvements in animal welfare at slaughter. Improvements, therefore, could be reliant upon potential legislative changes and/or educational or even financial encouragement from external organisations or governmental bodies, such as the Food Standards Agency in the UK.

Surprisingly, overall demographic factors, particularly participants’ professional experience seemed not to impact OV responses regarding job satisfaction or animal welfare at slaughter. This shows job satisfaction levels and views on animal welfare at slaughter to be largely consistent across the profession. This is potentially of concern since such consistency in opinion would suggest these may be held over a significant period of time, increasing the likelihood of burn-out and mental health conditions associated with the OV profession. Moreover, the majority of responding professionals were satisfied with the training they received in support of their role as an OV, a finding in direct contrast to that of Väärikkälä *et al.* (2020) and Gomez-Neves *et al.* (2023) who found that OVs had expressed the need for more training, particularly as regards communication and food hygiene. Further, there was a trend towards female respondents showing less satisfaction with training compared to their male counterparts. Indeed, this could indicate a potential gender bias in opinion on OV training, though this was not reported in previous research and would merit future research to assess potential motivations underlying such differences. Ultimately, as the legal representatives of animals at slaughter, it is imperative that OVs feel confident that the training they have received is fit for purpose and facilitates the decision-making processes required to take prompt legal action in food safety and animal welfare breaches. If there are potential risk factors, such as demographic features of OVs, then these should be identified and mitigated to harmonise the profession as much as possible, and future research may provide the means for understanding such complexities.

In this study, CCTV was reported to have contributed towards a reduction in animal welfare non-compliances according to OVs. Indeed, CCTV was introduced as a mandatory requirement in slaughterhouses as a means of improving animal welfare through transparency, as it can be used as evidence to support animal welfare cases (The Mandatory Use of Closed Circuit Television in Slaughterhouses [England] Regulations 2018). While our study only assesses OVs’ perceptions on CCTV as a refinement tool for improving animal welfare at slaughter, this is still promising since it suggests OVs are witnessing less animal welfare insults as a result of CCTV. It would be relevant to confirm the extent to which these findings translate into an actual reduction in the reporting of non-compliances in official reports. Another area of potential interest for future research would be to examine which types of non-compliances have reduced as a result of this mandatory requirement in slaughterhouses. In particular, whether it has reduced the reporting of non-compliances associated with pre-slaughter operations and/or on-farm welfare infringements, as this has implications not only for animal welfare but also meat quality and public health safety.

### Study limitations

There were several limitations identified in this study. Firstly, this is a case study of the UK and ROI, therefore in order to gain a better understanding of general trends across the profession, further studies are necessary including data from OVs throughout different countries. Secondly, the findings obtained were self-reported in a closed-ended questionnaire which did not allow for ideas, thoughts or feelings on a particular topic to be expanded upon. This could be achieved in future research using focus groups and undertaking a thematic analysis. Thirdly, OVs were asked their opinion on whether ritual slaughter without stunning should be banned without taking their practical experience with religious slaughter into account. It would have been beneficial to have acquired further information on what type of abattoir each OV worked in, as this could have offered further insight into how this method of slaughter affects professionals’ job satisfaction based on professionals’ experience with these methods. Fourthly, a larger sample size may have provided greater insight into general trends, particularly more participants from ROI, Northern Ireland and Wales, which were less represented in this study. And, finally, in this study, the salary of the OVs was unknown, yet salary is generally considered to influence job satisfaction (Kersebohm *et al.* 2017; Hagen *et al.* 2020), so this could have provided further insight into how this particular aspect could influence job satisfaction. In particular, further research is needed to elucidate how job dissatisfaction impacts the performance and emotional well-being of OVs and how this affects the quality of welfare inspections.

### Animal welfare implications

The observation of animal welfare insults has a detrimental effect on the job satisfaction of OVs throughout the UK and ROI, and the day-to-day reality of the job entails encountering practices that conflict with their professional ethics principles. This highlights a need for the impacts of animal welfare insults at slaughter to be reduced. Religious slaughter, in particular, impinges on the job satisfaction and emotional well-being of OVs. Further, the finding that at least a third of our participating professionals experienced threatening situations in the workplace is hugely concerning since it poses a significant risk to the well-being of OVs working to preserve public health. It is possible that closer collaboration with work colleagues could engender greater support when conflicts arise in the workplace. However, it is also essential for OVs to receive further training on decision-making when animal welfare insults arise and on effective communication skills to avoid a breakdown of the FBO-OV relationship. Inevitably this breakdown results in further social isolation in the slaughterhouse and experiencing loneliness in the workplace. It is possible that revisions to the national legislation on animal welfare at slaughter could lead to a drastic improvement in the satisfaction and commitment of OVs in the workplace. In particular, the implementation of more specific requirements, such as those stipulated by WATOK would be highly beneficial, with them perhaps even extended to other pre-slaughter operations that occur prior to animals reaching the slaughterhouse. An example of this would be to have CCTV deployed during catching, on-farm, and in the transport vehicle to monitor insults to welfare during these stages of the proceedings. This would also ensure further evidence in support of insults to animal welfare detected on the animal’s arrival at the slaughterhouse. Collectively, future efforts targeted at OV training, and application of more stringent measures when welfare breaches are detected could contribute both to improving animal welfare at slaughter and potentially job satisfaction for the OV.

## Supporting information

O’Connor and Gouveia supplementary material O’Connor and Gouveia supplementary material

## Supplementary material

The supplementary material for this article can be found at http://doi.org/10.1017/awf.2024.43.
