# Supplementary material for: Job satisfaction and animal welfare at slaughter: A survey of Official Veterinarians in the United Kingdom and Republic of Ireland
Source: Anim Welf. 2025 Jan 20;34:e7. doi: 10.1017/awf.2024.43 (PMC11810507; doi:10.1017/awf.2024.43)
Supplement: O’Connor and Gouveia supplementary material [file S0962728624000435sup001.pdf]

Table S1. General OV perceptions on job satisfaction and animal welfare at slaughter

		Number and percentage of respondents				
**(a) OV questions on overall job satisfaction**	**Total n (%)**	**Very satisfied**	**Somewhat satisfied**	**Neutral**	**Somewhat dissatisfied**	**Very dissatisfied**
Satisfaction with prior OV training	113 (100%)	56 (49.6%)	36 (31.9%)	12 (10.6%)	6 (5.3%)	3 (2.7%)
		**Very committed**	**Somewhat committed**	**Not committed**		
Level of commitment	112 (100%)	91 (81.3%)	20 (17.9%)	1 (0.9%)		
		**Optimal**	**Reasonable**	**Unsatisfactory**		
Perceived work life balance	113 (100%)	27 (23.9%)	70 (61.9%)	16 (14.2%)		
		**Never**	**Sometimes**	**Often**	**Always**	
Experiencing loneliness at work	113 (100%)	38 (33.6%)	46 (40.7%)	22 (19.5%)	7 (6.2%)	
Sleeping disorders due to work		34 (30.1%)	54 (47.8%)	21 (18.6%)	4 (3.5%)	
		**Yes**	**No**			
Threatening situations at work in the past 12 months	113 (100%)	36 (31.9%)	77 (68.1%)			
**(b) OV questions on animal welfare at slaughter**		**Never**	**Sometimes**	**Often**	**Always**	
Job satisfaction impacted by animal welfare incidents	113 (100%)	25 (22.1%)	45 (39.8%)	35 (31.0%)	8 (7.1%)	
		**Strongly disagree**	**Disagree**	**Neutral**	**Agree**	**Strongly agree**
Administration processes time consuming and interfering with animal welfare inspections	113 (100%)	5 (4.4%)	15 (13.3%)	37 (32.7%)	39 (34.5%)	17 (15.0%)
Satisfaction that FBO has a suitable system for protection of animal welfare	113 (100%)	2 (1.8%)	10 (8.8%)	9 (8.0%)	46 (40.7	46 (40.7%)
Ability to communicate welfare concerns to FBO and achieve compliance	113 (100%)	1 (0.9%)	1 (0.9%)	12 (10.6%)	59 (52.2%)	40 (35.4%)
Ritual slaughter should be banned in the UK and Ireland	113 (100%)		1 (0.9%)	15 (13.3%)	20 (17.7%)	75 (66.4%)

CCTV has reduced the incidence of animal welfare non-compliances?	113 (100%)	2 (1.8%)	5 (4.4%)	27 (23.9%)	43 (38.1%)	36 (31.9%)	
		**Financial constraints**	**Attitudes/willingness of FBO**	**Lack of legislative protection**	**All of the above**	**Other**	
Greatest barrier for improving animal welfare standards at slaughter	113 (100%)	20 (17.7%)	44 (38.9%)	5 (4.4%)	34 (30.1%)	10 (8.8%)	
		**Unloading**	**Lairage**	**Movement and restraint**	**Stunning**	**Bleeding**	**More than one option**
Area(s) of the slaughterhouse where most prominent welfare issues/concerns for animals occur	113 (100%)	33 (29.2%)	19 (16.8%)	19 (16.8%)	14 (12.4%)	4 (3.5%)	24 (21.2%)

Table S2. Statistical associations between age and OV responses

		Number and percentage of respondents						
**(a) OV questions on overall job satisfaction**	**Age category**	**Very satisfied**	**Somewhat satisfied**	**Neutral**	**Somewhat dissatisfied**	**Very dissatisfied**	**Test statistic**	***P*-value**
Satisfaction with prior OV training	Up to 30yrs	4 (30.8%)	6 (46.2)	1 (7.7%)	1 (7.7%)	1 (7.7%)		
	31-40yrs	17 (42.2%)	11 (30.6%)	5 (13.9)	3 (8.3%)	0 (0%)		
	41-50yrs	17 (42.5%)	15 (37.5%)	4 (10.0%)	2 (5.0%)	2 (5.0%)		
	51-60yrs	11 (78.6%)	3 (21.4%)	0 (0%)	0 (0%)	0 (0%)		
	61 yrs+	7 (70.0%)	1 (2.8)	2 (20.0%)	0 (0%)	0 (0%)		
							χ^2 (4) = 8.9	P = 0.07
		**Very committed**	**Somewhat committed**	**Not committed**				
Level of commitment	Up to 30yrs	10 (76.9%)	2 (15.4%)	1 (7.7)				
	31-40yrs	30 (85.7%)	5 (14.3%)	0 (0%)				
	41-50yrs	32 (80.0%)	8 (20.0%)	0 (0%)				
	51-60yrs	11 (78.6%)	3 (21.4)	0 (0%)				
	61 yrs+	8 (80%)	2 (20.0%)	0 (0%)				
							χ^2(4) = 0.8	P = 0.934
		**Optimal**	**Reasonable**	**Unsatisfactory**				
Perceived work life balance	Up to 30yrs	0 (0%)	8 (61.6%)	5 (38.5%)				
	31-40yrs	7 (19.4%)	24 (66.6%)	5 (13.9%)				
	41-50yrs	10 (25.0%)	26 (65.0%)	4 (10.0%)				

	51-60yrs	3 (21.4%)	9 (64.3%)	2 (14.3%)			
	61 yrs+	7 (70.0%)	3 (30.0%)	0 (0%)			
						χ^2 (4) = 18.5	P < 0.001
		**Never**	**Sometimes**	**Often**	**Always**		
Experiencing loneliness at work	Up to 30yrs	1 (7.7%)	4 (30.8%)	5 (38.5%)	3 (23.1%)		
	31-40yrs	12 (33.3%)	17 (47.2%)	6 (16.7%)	1 (2.8%)		
	41-50yrs	12 (30.0%)	18 (45.0%)	8 (20.0%)	2 (5.0%)		
	51-60yrs	7 (18.4%)	3 (21.4%)	3 (21.4%)	1 (7.1%)		
	61 yrs+	6 (60.0%)	4 (40.0%)	0 (0%)	0 (0%)		
						χ^2 (4) = 14.2	P = 0.007
Sleeping disorders due to work	Up to 30yrs	4 (30.8%)	3 (23.1%)	4 (30.8%)	2 (15.4%)		
	31-40yrs	11 (30.6%)	16 (44.4%)	9 (25.0%)	0 (0%)		
	41-50yrs	8 (20.0%)	26 (65.0%)	5 (12.5%)	1 (2.5%)		
	51-60yrs	7 (50.0%)	3 (21.4%)	3 (21.4%)	1 (7.1%)		
	61 yrs+	4 (40.0%)	6 (60.0%)	0 (0%)	0 (0%)		
						χ^2 (4) = 3.9	P = 0.408
		**Yes**	**No**				
Threatening situations at work in the past 12 months	Up to 30yrs	6 (46.2%)	7 (53.8%)				
	31-40yrs	13 (36.1%)	23 (63.9%)				
	41-50yrs	11 (27.5%)	29 (72.5%)				
	51-60yrs	4 (28.6%)	10 (71.4%)				
	61 yrs+	4 (28.6%)	10 (71.4%)				
						χ^2 (4) = 2.6	P = 0.628

**(b) OV questions on animal welfare at slaughter**		**Never**	**Sometimes**	**Often**	**Always**			
Job satisfaction impacted by animal welfare incidents	Up to 30yrs	2 (15.4%)	8 (61.5%)	3 (23.1%)	0 (0%)			
	31-40yrs	7 (19.4%)	16 (44.4%)	13 (36.1%)	0 (0%)			
	41-50yrs	7 (17.5%)	12 (30.0%)	16 (40.0%)	5 (12.5%)			
	51-60yrs	6 (42.9%)	4 (28.6%)	2 (14.3%)	2 (14.3%)			
	61 yrs+	3 (30.0%)	5 (50.0%)	1 (10.0%)	1 (10.0%)			
							χ^2 (4) = 5.7	*P* = 0.223
		**Strongly disagree**	**Disagree**	**Neutral**	**Agree**	**Strongly agree**		
Administration processes time consuming and interfering with animal welfare inspections	Up to 30yrs	2 (15.4%)	3 (23.1%)	7 (53.8%)	1 (7.7%)	0 (0%)		
	31-40yrs	5 (13.9%)	14 (38.9%)	12 (33.3%)	4 (11.1%)	1 (2.8%)		
	41-50yrs	8 (20.0%)	14 (35.0%)	10 (25.0%)	7 (17.5%)	1 (2.5%)		
	51-60yrs	1 (7.1%)	4 (28.6%)	5 (35.7%)	2 (14.3%)	2 (14.3%)		
	61 yrs+	1 (10.0%)	4 (40.0%)	3 (30.0%)	1 (10.0%)	1 (10.0%)		
							χ^2 (4) = 2.4	*P* = 0.660
Satisfaction that FBO has a suitable system for protection of animal welfare	Up to 30yrs	0 (0%)	0 (0%)	2 (15.4%)	9 (69.2%)	2 (15.4%)		
	31-40yrs	1 (2.8%)	5 (13.9%)	2 (5.6%)	15 (41.7%)	16 (40%)		
	41-50yrs	1 (2.5%)	4 (10.0%)	4 (10.0%)	15 (37.5%)	16 (40%)		
	51-60yrs	0 (0%)	0 (0%)	1 (7.1%)	4 (28.6%)	9 (64.3%)		
	61 yrs+	0 (0%)	1 (10.0%)	0 (0%)	3 (30.0%)	6 (60.0%)		
							χ^2 (4) = 6.8	*P* = 0.146
Ability to communicate welfare concerns to FBO and achieve compliance	Up to 30yrs	1 (7.7%)	0 (0%)	1 (7.7%)	7 (53.8%)	12 (33.3%)		

	31-40yrs	0 (0%)	0 (0%)	3 (8.3%)	21 (58.3%)	12 (33.3%)		
	41-50yrs	0 (0%)	1 (2.5%)	6 (15.0%)	19 (47.5)	14 (35.0%)		
	51-60yrs	0 (0%)	0 (0%)	1 (7.1%)	7 (50.0%)	6 (42.9%)		
	61 yrs+	0 (0%)	0 (0%)	1 (10.0%)	5 (50.0%)	4 (40.0%)		
							χ^2 (4) = 1.06	*P* = 0.901
Ritual slaughter should be banned in the UK and Ireland	Up to 30yrs		1 (7.1%)	3 (23.1%)	1 (7.7%)	8 (61.5%)		
	31-40yrs		1 (2.8%)	7 (19.4%)	8 (22.2%)	20 (55.6%)		
	41-50yrs		0 (0%)	4 (10.0%)	9 (22.5%)	27 (67.5%)		
	51-60yrs		0 (0%)	0 (0%)	2 (14.3%)	12 (85.7%)		
	61 yrs+		1 (10.0%)	1 (10.0%)	0 (0%)	8 (80.0%)		
							χ^2 (4) = 5.7	*P* = 0.225
CCTV has reduced the incidence of animal welfare non-compliances?	Up to 30yrs	0 (0.0%)	0 (0.0%)	4 (30.8%)	5 (38.5%)	4 (30.8%)		
	31-40yrs	2 (5.6%)	1 (2.8%)	5 (13.9%)	16 (44.4%)	12 (33.3%)		
	41-50yrs	0 (0.0%)	2 (5.0%)	12 (30.0%)	13 (32.5%)	13 (32.5%)		
	51-60yrs	0 (0.0%)	0 (0.0%)	2 (14.3%)	7 (50.0%)	5 (35.7%)		
	61 yrs+	0 (0.0%)	2 (20.0%)	4 (40.0%)	2 (20.0%)	2 (20.0%)		
							χ^2 (4) = 4.4	*P* = 0.352
		**Financial constraints**	**Attitudes/ willingness of FBO**	**Lack of legislative protection**	**All of the above**	**Other**		
Greatest barrier for improving animal welfare standards at slaughter	Up to 30yrs	2 (15.4%)	6 (46.2%)	1 (7.7%)	2 (15.4%)	2 (15.4%)		
	31-40yrs	7 (19.4%)	9 (25.0%)	1 (2.8%)	18 (50.0%)	1 (2.8%)		
	41-50yrs	8 (20.0%)	17 (42.5%)	1 (2.5%)	11 (27.5%)	3 (7.5%)		
	51-60yrs	2 (14.3%)	8 (57.1%)	1 (7.1%)	1 (7.1%)	2 (14.3%)		

	61 yrs+	1 (10.0%)	4 (40.0%)	1 (10.0%)	2 (20.0%)	2 (20.0%)			
								χ^2 (16) = 18.4	*P* = 0.302
		**Unloading**	**Lairage**	**Movement and restraint**	**Stunning**	**Bleeding**	**More than one option**		
Area(s) of the slaughterhouse where most prominent welfare issues/concerns for animals occur	Up to 30yrs	4 (30.8%)	1 (7.7%)	1 (7.7%)	3 (23.1%)	2 (15.4%)	2 (15.4%)		
	31-40yrs	6 (16.7%)	6 (16.7%)	6 (16.7%)	6 (16.7%)	0 (0%)	12 (33.3%)		
	41-50yrs	10 (25.0%)	10 (25.0%)	6 (15.0%)	5(12.5%)	1 (2.5%)	8 (20.0%)		
	51-60yrs	7 (50.0%)	2 (14.3%)	4 (28.6%)	0 (0%)	0 (0%)	1 (10.0%)		
	61 yrs+	6 (60.0%)	0 (0%)	2 (20.0%)	0 (0%)	1 (10.0%)	1 (10.0%)		
								χ^2 (20) = 40.0	*P* = 0.056

Table S3. Statistical associations between professional experience and OV responses

		Number and percentage of respondents						
**(a) OV questions on overall job satisfaction**	**Job Experience category**	**Very satisfied**	**Somewhat satisfied**	**Neutral**	**Somewhat dissatisfied**	**Very dissatisfied**		***P*-value**
Satisfaction with prior OV training	Up to 5yrs	27 (44.3%)	22 (36.1%)	6 (9.8%)	4 (6.6%)	2 (3.3%)		
	6-9 yrs	6 (40.0%)	4 (26.7%)	4 (26.7%)	0 (0%)	1 (6.7%)		
	10+ years	23 (62.2%)	10 (27.0%)	2 (5.4%)	2 (5.4%)	0 (0%)		
							χ^2 (2) = 4.2	*P* = 0.120
		**Very committed**	**Somewhat committed**	**Not committed**				
Level of commitment	Up to 5yrs	49 (80.3%)	11 (18.0%)	1 (1.6%)				
	6-9 yrs	14 (93.3%)	1 (6.7%)	0 (0%)				
	10+ years	28 (77.8%)	8 (22.2%)	0 (0%)				
							χ^2 (2) = 1.7	*P* = 0.421
		**Optimal**	**Reasonable**	**Unsatisfactory**				
Perceived work life balance	Up to 5yrs	15 (24.6%)	35 (57.4%)	11 (18.0%)				
	6-9 yrs	3 (20.0%)	8 (53.3%)	4 (26.7%)				
	10+ years	9 (24.3%)	27 (73.0%)	1 (2.7%)				
							χ^2 (2) = 2.3	*P* = 0.310
		**Never**	**Sometimes**	**Often**	**Always**			
Experiencing loneliness at work	Up to 5yrs	20 (32.8%)	24 (39.3%)	14 (23.0%)	3 (4.9%)			
	6-9 yrs	3 (20.0%)	9 (60.0%)	0 (0.0%)	3 (20.0%)			
	10+ years	15 (40.5%)	13 (35.1%)	8 (21.6%)	1 (2.7%)			
							χ^2 (2) = 1.2	*P* = 0.559
Sleeping disorders due to work	Up to 5yrs	18 (29.5%)	29 (47.5%)	11 (18.0%)	3 (4.9%)			

	6-9 yrs	3 (20.0%)	9 (60.0%)	3 (20.0%)	0 (0%)			
	10+ years	13 (35.1%)	16 (43.2%)	7 (18.9%)	1 (2.7%)			
							χ^2 (2) = 0.5	*P* = 0.796
		**Yes**	**No**					
Threatening situations at work in the past 12 months	Up to 5yrs	20 (32.8%)	41 (67.2%)					
	6-9 yrs	5 (33.3%)	10 (66.7%)					
	10+ years	11 (29.7%)	26 (70.3%)					
							χ^2 (2) = 0.1	*P* = 0.943
**(b) OV questions on animal welfare at slaughter**		**Never**	**Sometimes**	**Often**	**Always**			
Job satisfaction impacted by animal welfare incidents	Up to 5yrs	13 (23.1%)	26 (42.6%)	19 (31.1%)	3 (4.9%)			
	6-9 yrs	2 (13.3%)	7 (46.7%)	5 (33.3%)	1 (6.7%)			
	10+ years	10 (27.0%)	12 (32.4%)	11 (29.7%)	4 (10.8%)			
							χ^2 (2) = 0.3	*P* = 0.869
		**Strongly disagree**	**Disagree**	**Neutral**	**Agree**	**Strongly agree**		
Administration processes time consuming and interfering with animal welfare inspections	Up to 5yrs	1 (1.6%)	8 (13.1%)	24 (39.3%)	20 (32.8%)	8 (13.1%)		
	6-9 yrs	1 (6.7%)	2 (13.3%)	6 (40.0%)	2 (13.3%)	4 (26.7%)		
	10+ years	3 (8.1%)	5 (13.5%)	7 (18.9%)	17 (45.9%)	5 (13.5%)		
							χ^2 (2) = 0.2	*P* = 0.899
Satisfaction that FBO has a suitable system for protection of animal welfare	Up to 5yrs	1 (1.6%)	7 (11.5%)	5 (8.2%)	27 (44.3%)	21 (34.4%)		
	6-9 yrs	5 (33.3%)	5 (33.3%)	3 (20.0%)	1 (6.7%)	1 (6.7%)		
	10+ years	20 (54.1%)	14 (37.8%)	1 (2.7%)	2 (5.4%)	0 (0%)		
							χ^2 (2) = 5.7	*P* = 0.060

Ability to communicate welfare concerns to FBO and achieve compliance	Up to 5yrs	1 (1.6%)	0 (0%)	9 (14.8%)	30 (49.2%)	21 (34.4%)		
	6-9 yrs	0 (0%)	1 (6.7%)	1 (6.7%)	11 (73.3%)	2 (13.3%)		
	10+ years	0 (0%)	0 (0%)	2 (5.4%)	18 (48.6%)	17 (45.9%)		
							χ^2 (2) = 5.2	*P* = 0.076
Ritual slaughter should be banned in the UK and Ireland	Up to 5yrs		1 (1.6%)	10 (16.4%)	11 (18.0%)	39 (63.9%)		
	6-9 yrs		2 (13.3%)	2 (13.3%)	3 (20.0%)	8 (53.3%)		
	10+ years		0 (0%)	3 (8.1%)	6 (16.2%)	28 (75.7%)		
							χ^2 (2) = 3.6	*P* = 0.166
CCTV has reduced the incidence of animal welfare non-compliances?	Up to 5yrs	1 (1.6%)	3 (4.9%)	14 (23.0%)	22 (36.1%)	21 (34.4%)		
	6-9 yrs	0 (0%)	1 (6.7%)	5 (33.3%)	6 (40.0%)	3 (20.0%)		
	10+ years	1 (2.7%)	1 (2.7%)	8 (21.6%)	15 (40.5%)	12 (34.4%)		
							χ^2 (2) = 1.2	*P* = 0.550
		**Financial constraints**	**Attitudes/ willingness of FBO**	**Lack of legislative protection**	**All of the above**	**Other**		
Greatest barrier for improving animal welfare standards at slaughter	Up to 5yrs	10 (16.4%)	19 (31.1%)	2 (3.3%)	24 (39.3%)	6 (9.8%)		
	6-9 yrs	0 (0%)	8 (53.3%)	1 (6.7%)	4 (26.7%)	2 (13.3%)		
	10+ years	10 (27.0%)	17 (45.9%)	2 (5.4%)	6 (16.2%)	2 (5.4%)		
							χ^2 (8) = 12.3	P = 0.141
		**Unloading**	**Lairage**	**Movement and restraint**	**Stunning**	**Bleeding**	**More than one option**	
Area(s) of the slaughterhouse where most prominent welfare issues/concerns for animals occur	Up to 5yrs	14 (23.0%)	10 (16.4%)	10 (16.4%)	10 (16.4%)	4 (6.6%)	13 (21.3%)	
	6-9 yrs	4 (26.7%)	3 (20.0%)	2 (13.3%)	2 (13.3%)	0 (0%)	4 (26.7%)	
	10+ years	15 (40.5%)	6 (16.2%)	7 (18.9%)	2 (5.4%)	0 (0%)	7 (18.9%)	
							χ^2 (10) = 8.8	*P* = 0.555

Table S4. Statistical associations between gender and OV responses

		Number and percentage of participants						
**(a) OV questions on overall job satisfaction**	**Job Experience category**	**Very satisfied**	**Somewhat satisfied**	**Neutral**	**Somewhat dissatisfied**	**Very dissatisfied**	**Test statistic**	***P*-value**
Satisfaction with prior OV training	Male	38 (57.6%)	18 (27.3%)	6 (9.1%)	1 (1.5%)	3 (4.5%)		
	Female	18 (38.3%)	18 (38.3%)	6 (12.8%)	5 (10.6%)	0 (0%)		
							U = 1247.5	P = 0.054
		**Very committed**	**Somewhat committed**	**Not committed**				
Level of commitment	Male	52 (78%)	13 (19.7%)	1 (1.5%)				
	Female	39 (84.8%)	7 (15.2%)	0 (0%)				
							U = 1423.5	P = 0.409
		**Optimal**	**Reasonable**	**Unsatisfactory**				
Perceived work life balance	Male	21 (31.8%)	33 (50.0%)	12 (18.2%)				
	Female	6 (12.8%)	37 (78.7%)	4 (8.5%)				
							U = 1411.5	P = 0.347
		**Never**	**Sometimes**	**Often**	**Always**			
Experiencing loneliness at work	Male	25 (37.9%)	25 (37.9%)	11 (16.7%)	5 (7.6%)			
	Female	13 (27.7%)	21 (44.7%)	11 (23.4%)	2 (4.3%)			
							U = 1411	P = 0.399
Sleeping disorders due to work	Male	22 (33.3%)	31 (47.0%)	11 (16.7%)	2 (3.0%)			
	Female	12 (25.5%)	23 (48.9%)	10 (21.3%)	2 (4.3%)			
							U = 1392.5	P = 0.319
		**Yes**	**No**					
Threatening situations at work in the past 12 months	Male	20 (30.3%)	46 (69.7%)					
	Female	16 (34.0%)	31 (66.0%)					
							$\chi^2(1) = 0.18$	P = 0.674
**(b) OV questions on animal welfare at slaughter**		**Never**	**Sometimes**	**Often**	**Always**			
Job satisfaction impacted by animal welfare incidents	Male	14 (21.2%)	24 (36.4%)	20 (30.3%)	8 (12.1%)			
	Female	11 (23.4%)	21 (44.7%)	15 (31.9%)	0 (0%)			

								U = 1343.5	*P* = 0.201
		**Strongly disagree**	**Disagree**	**Neutral**	**Agree**	**Strongly agree**			
Administration processes time consuming and interfering with animal welfare inspections	Male	3 (4.5%)	6 (9.1%)	19 (28.8%)	24 (36.4%)	14 (21.2%)			
	Female	2 (4.3%)	9 (19.1%)	18 (38.3%)	15 (31.9%)	3 (6.4%)			
								U = 1167	*P* = 0.020
Satisfaction that FBO has a suitable system for protection of animal welfare	Male	0 (0%)	6 (9.1%)	7 (10.6%)	24 (36.4%)	29 (43.9%)			
	Female	2 (4.3%)	4 (8.5%)	2 (4.3%)	22 (46.8%)	17 (36.2%)			
								U = 1456.5	*P* = 0.554
Ability to communicate welfare concerns to FBO and achieve compliance	Male	1 (1.5%)	1 (1.5%)	7 (10.6%)	37 (56.1%)	20 (30.3%)			
	Female	0 (0%)	0 (0%)	5 (10.6%)	22 (46.8%)	20 (42.6%)			
								U = 1349.5	*P* = 0.193
Ritual slaughter should be banned in the UK and Ireland	Male		3 (4.5%)	10 (15.2%)	15 (22.7%)	38 (57.6%)			
	Female		37 (78.7%)	5 (10.6%)	5 (10.6%)	0 (0%)			
								U = 1220.5	*P* = 0.021
CCTV has reduced the incidence of animal welfare non-compliances?	Male	1 (1.5%)	4 (6.1%)	19 (28.8%)	20 (30.3%)	22 (33.3%)			
	Female	1 (2.1%)	1 (2.1%)	8 (17.0%)	23 (48.9%)	14 (29.8%)			
								U = 1430.5	*P* = 0.459
		**Financial constraints**	**Attitudes/ willingness of FBO**	**Lack of legislative protection**	**All of the above**	**Other**			
Greatest barrier for improving animal welfare standards at slaughter	Male	11 (16.7%)	30 (45.5%)	2 (3.0%)	18 (27.3%)	5 (7.6%)			
	Female	9 (19.1%)	14 (29.8%)	3 (6.4%)	16 (34.0%)	5 (10.6%)			
								χ^2 (4) = 3.2	*P* = 0.520
		**Unloading**	**Lairage**	**Movement and restraint**	**Stunning**	**Bleeding**	**More than one option**		
Area(s) of the slaughterhouse where most prominent welfare issues/concerns for animals occur	Male	21 (31.8%)	11 (16.7%)	9 (13.6%)	6 (9.1%)	2 (3.0%)	17 (25.8%)		

	Female	12 (25.5%)	8 (17.0%)	10 (21.3%)	8 (17.0%)	2 (4.3%)	7 (14.9%)	χ^2 (5) = 4.4	P = 0.499

Table S5. Statistical associations between species slaughtered and OV responses

		Number and percentage of participants						
**(a) OV questions on overall job satisfaction**	**Species slaughtered**	**Very satisfied**	**Somewhat satisfied**	**Neutral**	**Somewhat dissatisfied**	**Very dissatisfied**	**Test statistic**	***P*-value**
Satisfaction with prior OV training	Ruminants	34 (48.6%)	24 (34.3%)	6 (8.6%)	4 (5.7%)	2 (2.9%)		
	Pigs	11 (50.0%)	5 (22.7%)	4 (18.2%)	2 (9.1%)	0 (0%)		
	Poultry	11 (52.4%)	7 (33.3%)	2 (9.5%)	0 (0%)	1 (4.8%)		
							χ^2 (2) = 0.2	P = 0.890
		**Very committed**	**Somewhat committed**	**Not committed**				
Level of commitment	Ruminants	53 (76.8%)	15 (21.7%)	1 (1.4%)				
	Pigs	19 (86.4%)	3 (13.6%)	0 (0%)				
	Poultry	19 (90.5%)	2 (9.5%)	0 (0%)				
							χ^2 (2) = 2.5	P = 0.289
		**Optimal**	**Reasonable**	**Unsatisfactory**				
Perceived work life balance	Ruminants	20 (28.6%)	42 (60.0%)	8 (11.4%)				
	Pigs	3 (13.6%)	13 (59.1%)	6 (27.3%)				
	Poultry	4 (19.0%)	15 (71.4%)	2 (9.5%)				
							χ^2 (2) = 4.1	P = 0.127
		**Never**	**Sometimes**	**Often**	**Always**			
Experiencing loneliness at work	Ruminants	28 (40.0%)	26 (37.1%)	11 (15.7%)	5 (7.1%)			
	Pigs	6 (27.3%)	11 (50.0%)	5 (22.7%)	0 (0%)			
	Poultry	4 (19.0%)	9 (42.9%)	6 (28.6%)	2 (9.5%)			
							χ^2 (2) = 3.5	P = 0.174
Sleeping disorders due to work	Ruminants	23 (32.9%)	33 (47.1%)	13 (18.6%)	1 (1.4%)			
	Pigs	6 (27.3%)	11 (50.0%)	3 (13.6%)	2 (9.1%)			
	Poultry	5 (23.8%)	10 (47.6%)	5 (23.8%)	1 (4.8%)			
							χ^2 (2) = 1.1	P = 0.561
		**Yes**	**No**					
Threatening situations at work in the past 12 months	Ruminants	26 (37.1%)	44 (62.9%)					
	Pigs	5 (22.7%)	17 (77.3%)					
	Poultry	5 (23.8%)	16 (20.8%)					
							χ^2 (2) = 2.3	P = 0.305

**(b) OV questions on animal welfare at slaughter**		**Never**	**Sometimes**	**Often**	**Always**			
Job satisfaction impacted by animal welfare incidents	Ruminants	16 (22.9%)	34 (48.6%)	15 (21.4%)	5 (7.1%)			
	Pigs	5 (22.7%)	7 (31.8%)	10 (45.5%)	0 (0%)			
	Poultry	4 (19%)	4 (19%)	10 (47.6%)	3 (14.3%)			
							χ^2 (2) = 4.5	P = 0.108
		**Strongly disagree**	**Disagree**	**Neutral**	**Agree**	**Strongly agree**		
Administration processes time consuming and interfering with animal welfare inspections	Ruminants	5 (7.1%)	8 (11.4%)	25 (35.7%)	21 (30.0%)	11 (15.7%)		
	Pigs	0 (0%)	3 (13.6%)	9 (40.9%)	8 (36.4%)	2 (9.1%)		
	Poultry	0 (0%)	4 (19.0%)	3 (14.3%)	10 (47.6%)	4 (19.0%)		
							χ^2 (2) = 1.5	P = 0.459
FBO has a suitable system for protection of animal welfare	Ruminants	1 (1.4%)	5 (7.1%)	6 (8.6%)	28 (40.0%)	30 (42.9%)		
	Pigs	1 (4.5%)	3 (13.6%)	1 (4.5%)	8 (36.4%)	9 (40.9%)		
	Poultry	0 (0%)	2 (9.5%)	2 (9.5%)	10 (47.6%)	7 (33.3%)		
							χ^2 (2) = 0.5	P = 0.767
Ability to communicate welfare concerns to FBO and achieve compliance	Ruminants	1 (1.4%)	0 (0%)	8 (11.4%)	34 (48.65%)	27 (38.6%)		
	Pigs	0 (0%)	0 (0%)	3 (13.6%)	13 (59.1%)	6 (27.3%)		
	Poultry	1 (0.9%)	1 (0.9%)	12 (10.6%)	59 (52.2%)	40 (35.4%)		
							χ^2 (2) = 0.6	P = 0.727
Ritual slaughter should be banned in the UK and Ireland	Ruminants		2 (2.9%)	9 (12.9%)	10 (14.3%)	49 (70.0%)		
	Pigs			2 (9.1%)	6 (27.3%)	13 (59.1%)		
	Poultry							
							χ^2 (2) = 0.7	P = 0.686
CCTV has reduced the incidence of animal welfare non-compliances?	Ruminants	1 (1.4%)	3 (4.3%)	15 (21.4%)	24 (34.3%)	27 (38.6%)		
	Pigs	0 (0%)	1 (4.5%)	9 (40.9%)	10 (45.5%)	2 (9.1%)		
	Poultry	1 (4.8%)	1 (4.8%)	3 (14.3%)	9 (42.9%)	7 (33.3%)		
							χ^2 (2) = 5.6	P = 0.070

		**Financial constraints**	**Attitudes/ willingness of FBO**	**Lack of legislative protection**	**All of the above**	**Other**			
Greatest barrier for improving animal welfare standards at slaughter	Ruminants	12 (17.1%)	27 (38.6%)	3 (4.3%)	20 (28.6%)	8 (11.4%)			
	Pigs	3 (13.6)	9 (40.9%)	1 (4.5%)	7 (31.8%)	2 (9.1%)			
	Poultry	5 (23.8%)	8 (38.1%)	1 (4.8%)	7 (33.3%)	0 (0%)			
								χ^2 (8) = 3.2	P = 0.919
		**Unloading**	**Lairage**	**Movement and restraint**	**Stunning**	**Bleeding**	**More than one option**		
Area(s) of the slaughterhouse where most prominent welfare issues/concerns for animals occur	Ruminants	21 (30.0%)	13 (18.6%)	12 (17.1%)	9 (12.9%)	2 (2.9%)	13 (18.6%)		
	Pigs	10 (45.5%)	1 (4.5%)	4 (18.2%)	2 (9.1%)	0 (0%)	5 (22.7%)		
	Poultry	2 (9.5%)	5 (23.8%)	3 (14.3%)	3 (14.3%)	2 (9.5%)	6 (28.6%)		
								χ^2 (10) = 11.7	P = 0.309

Table S6. Planned comparisons for effects of age

	**Effect of age on work life balance**	
**Age category**	**Test statistic**	***P*-value**
Up to 30 years-31 to 40 years	$U = 148.5$	$P = 0.022$
Up to 30 years-41-50 years	$U = 146$	$P = 0.006$
Up to 30 years-51-60 years	$U = 57$	$P = 0.054$
Up to 30 years-60+ years	$U = 12$	$P < 0.001$
31-40 years-41-50 years	$U = 663$	$P = 0.479$
31-40 years-51-60 years	$U = 248$	$P = 0.928$
31-40 years-60+ years	$U = 81.5$	$P = 0.007$
41-50 years-51-60 years	$U = 262$	$P = 0.674$
41-50 years-60+ years	$U = 104$	$P = 0.008$
51-60 years-60+ years	$U = 33$	$P = 0.016$
	**Effect of age on loneliness at work**	
Up to 30 years-31 to 40 years	$U = 113.5$	$P = 0.004$
Up to 30 years-41-50 years	$U = 143$	$P = 0.011$
Up to 30 years-51-60 years	$U = 46.5$	$P = 0.025$
Up to 30 years-60+ years	$U = 15$	$P = 0.001$
31-40 years-41-50 years	$U = 672$	$P = 0.59$
31-40 years-51-60 years	$U = 235$	$P = 0.695$
31-40 years-60+ years	$U = 118$	$P = 0.072$
41-50 years-51-60 years	$U = 246$	$P = 0.477$
41-50 years-60+ years	$U = 120$	$P = 0.037$
51-60 years-60+ years	$U = 55$	$P = 0.331$
